# Acquired hemophilia a caused by complicated urinary tract infection due to flavobacterium odoratum: A case report and literature review

**DOI:** 10.1097/MD.0000000000050501

**Published:** 2026-09-04

**Authors:** Di Chen, Ping Yuan, Mingqiang Ren

**Affiliations:** a Department of Hematology, Affiliated Hospital of Zunyi Medical University, Zunyi, Guizhou, China.

**Keywords:** acquired hemophilia A, flavobacterium odoratum, infections

## Abstract

**Rationale:**

Acquired hemophilia A (AHA) is a rare bleeding disorder characterized by the development of autoantibodies directed against coagulation factor VIII (FVIII). Approximately 50% of cases are idiopathic, while the remaining are associated with autoimmune conditions, malignancies, or infections. To date, there have been no reported cases of AHA secondary to Flavobacterium odoratum (*Frullania odoratum*) infection.

**Patient concerns:**

A male patient underwent ureteroscopic lithotripsy with double-J stent placement. Postoperatively, he developed hematuria, a right medial thigh hematoma, fever, and abnormal coagulation parameters, including prolonged activated partial thromboplastin time (APTT).

**Diagnoses:**

The diagnosis of AHA was confirmed based on prolonged APTT, markedly reduced FVIII:C, and detection of a factor VIII inhibitor. Additionally, the patient had a complicated urinary tract infection caused by multidrug-resistant *F.odoratum*, which preceded the onset of AHA.

**Interventions:**

Doxycycline was administered to treat the catheter-associated urinary tract infection caused by *F.odoratum*. Hemostatic control was achieved with activated prothrombin complex concentrate. Immunosuppressive therapy for AHA included dexamethasone and cyclophosphamide.

**Outcomes:**

At discharge, bleeding had resolved, APTT normalized, FVIII activity returned to normal levels, the FVIII inhibitor was undetectable, and the infection was successfully controlled.

**Lessons:**

AHA is a rare but potentially life-threatening condition. Early recognition, prompt treatment, and identification of underlying triggers are crucial to reducing mortality and improving outcomes. This is the first reported case of AHA associated with *F. odoratum* infection. Clinicians should consider this pathogen in patients with long-term indwelling urinary catheters who present with unexplained infections or bleeding diatheses.

## 1. Introduction

Acquired hemophilia A (AHA) is a rare hemorrhagic disorder caused by the development of autoantibodies directed against coagulation factor VIII (FVIII), resulting in reduced FVIII activity. Clinically, it presents with sudden-onset and variable degrees of bleeding, including cutaneous, muscular, and visceral hemorrhages, with reported mortality rates ranging from 15% to 42%.^[1]^ Approximately 50% of AHA cases are associated with identifiable underlying conditions or etiologies, such as autoimmune diseases, malignancies, specific drug exposures, or infections^[2–7]^; therefore, early identification of potential triggering factors is critical. Flavobacterium odoratum (*F. odoratum*) is a Gram-negative opportunistic pathogen, with only around 30 documented cases worldwide.^[8]^ This organism is exceptionally rare and typically exhibits multidrug resistance, frequently leading to failure of empiric antimicrobial therapy. To date, there have been no reported cases of *F. odoratum* infection complicated by AHA. Herein, we present and summarize the diagnostic and therapeutic experience of a case of urinary tract infection secondary to *F. odoratum* that was complicated by AHA.

## 2. Case presentation

### 2.1. Basic information

A 50-year-old male was admitted on October 19, 2021, due to persistent hematuria, a right medial thigh hematoma, and fever lasting >10 days. Ten days prior to admission, he underwent ureteroscopic lithotripsy and double-J stent placement for ureteral calculi at an outside hospital. Postoperatively, he developed hematuria and a hematoma on the inner aspect of the right thigh, accompanied by fever peaking at 38.6°C. Serial coagulation tests revealed prolonged activated partial thromboplastin time (APTT). Despite intermittent fresh frozen plasma transfusions and empirical anti-infective treatment, his clinical condition did not improve significantly. Emergency laboratory investigations showed: white blood cell count, 10.51 × 10^9^/L, neutrophil count, 6.83 × 10^9^/L (reference range = 3.5~9.5 × 10^9^/L), hemoglobin, 63 g/L (reference range = 130~175g/L), and platelet count, 547 × 10^9^/L (reference range = 100~300 × 10^9^/L). The patient denied use of anticoagulants or other medications, had no history of childhood-onset recurrent spontaneous bleeding, and no prior diagnosis of malignancy or autoimmune disorders.

### 2.2. Physical examination

On admission, vital signs were as follows: body temperature, 36.7°C; pulse rate, 88 beats per minute; respiratory rate, 19 breaths per minute; and blood pressure, 139/85 mm Hg. The patient was alert and oriented. Physical examination revealed a 5 × 5 cm ecchymotic area in the right antecubital fossa, a 5 cm × 5 cm hematoma in the right lower quadrant of the abdomen, and an 8 cm × 9 cm bruise over the medial aspect of the right femoral region, all well-demarcated. The affected skin areas exhibited slightly elevated temperature, marked swelling, a smooth surface, and absence of fluctuation or ulceration. Limb sensation and motor function were intact, dorsalis pedis pulses were palpable bilaterally, and the indwelling urinary catheter was patent, draining continuous dark red urine. The remainder of the physical examination was unremarkable.

Auxiliary Examinations upon Admission: Serial routine blood tests after admission revealed a progressive decline in hemoglobin levels (HgBmin52g/L), with platelet count and platelet aggregation function within normal limits. Urinalysis showed urinary occult blood 3+, urinary protein 2+, 18,290 red blood cells, and 10 white blood cells per high-power field. Coagulation profiling demonstrated an elevated APTT of 113.1 seconds, while all other coagulation parameters were within reference ranges. Two sets of blood cultures for bacterial pathogens were negative. Factor VIII activity was markedly reduced at 0.7% (normal range: 70%–120%), consistent with severe deficiency. Antinuclear antibody testing was positive at a titer of 1:100 with a cytoplasmic granular pattern; however, further autoimmune evaluation, including anticardiolipin antibody, lupus anticoagulant, antinuclear antibody profile, immunoglobulin quantification, and other serological markers, revealed no abnormalities. Comprehensive metabolic assessments, including electrolytes, liver and renal function, lipid panel, blood glucose, myocardial enzyme spectrum, stool analysis, and screening for HIV, syphilis, hepatitis B and C, tumor markers, and thyroid function, were all within normal limits. Arteriovenous Doppler ultrasound of the right lower limb showed no evidence of thrombosis or vascular abnormalities. Chest CT scanning revealed no significant pulmonary or mediastinal findings. Abdominal CT imaging demonstrated left kidney enlargement with dilatation of the left renal pelvis and ureter, consistent with hydronephrosis, status post placement of a double-J stent in the left ureter, and a hyperdense patchy area in the right anterior abdominal wall suggestive of hematoma.

Diagnostic Approach: In middle-aged patients presenting with bleeding manifestations such as hematuria and right thigh hematoma, along with prolonged APTT and significantly decreased FVIII:C activity, hemophilia A should be considered initially. However, differentiation from other coagulation disorders: including congenital hemophilia, AHA, and von Willebrand disease: is essential.

The diagnostic approach consisted of 3 steps:

First, family history was assessed. No familial bleeding disorder was reported; however, hereditary coagulation defects could not be entirely excluded. Genetic testing for thrombosis- and coagulation-related conditions identified abnormalities in the CNVs region, but no mutations were detected in the introns of *F8* or inversion in intron 22, thereby ruling out congenital hemophilia.

Second, an APTT correction test was performed and yielded positive results (APTT: 102.50 seconds in patient plasma; 28.2 seconds in normal pooled plasma; 34.3 seconds in immediate 1:1 mix; and 48.90 seconds after 2-hour incubation). The von Willebrand factor antigen level was 137.20% (normal range: 42%–140.8% for blood type O; 66.1%–176.3% for others). Factor VIII inhibitor titer was elevated at 4.40 Bethesda units (BU) (normal: 0–0.6), while Factor IX inhibitor was negative. Based on these findings, a diagnosis of AHA was confirmed, and coagulation dysfunction due to congenital hemophilia or von Willebrand disease was excluded.

Third, the underlying etiology of AHA was investigated. The patient underwent ureteroscopic lithotripsy with double-J stent placement followed by prolonged catheterization, complicated by fever and hematuria. Urinalysis revealed pyuria, and 2 urine cultures were positive for multidrug-resistant *F.odoratum.* Therefore, this case of AHA may be associated with a complicated urinary tract infection caused by *F. odoratum*.

Treatment process and outcomes: The patient with AHA received intermittent infusions of fresh frozen plasma at a dose of 10 mL/kg, in combination with human prothrombin complex concentrate (50 IU/kg), intravenous cyclophosphamide (400 mg twice weekly), and dexamethasone (7.5 mg daily) to promote the clearance of factor VIII inhibitors. With regard to infectious complications, the patient was diagnosed with a urinary tract infection upon admission. Initial antimicrobial therapy with piperacillin-tazobactam and levofloxacin was ineffective. Repeated urine cultures identified a multidrug-resistant Bacteroides species as the causative pathogen. Consequently, antibiotic therapy was switched to oral doxycycline (100 mg every 12 hours) for a total duration of 2 weeks. On November 25, 2021, follow-up laboratory testing showed normalization of coagulation parameters, with factor VIII activity at 52.20% and undetectable levels of factor VIII inhibitor. Abdominal CT imaging revealed reduced left-sided urinary tract dilation compared to prior studies. Subsequent follow-up evaluations consistently demonstrated normalized APTT and sustained factor VIII activity within the normal reference range.

## 3. Key effect indicator

After admission, the patient’s APTT, FVIII activity, FVIII antibody, urine quantitative culture, and drug sensitivity test results were dynamically monitored during treatment.

**Figure 1.**
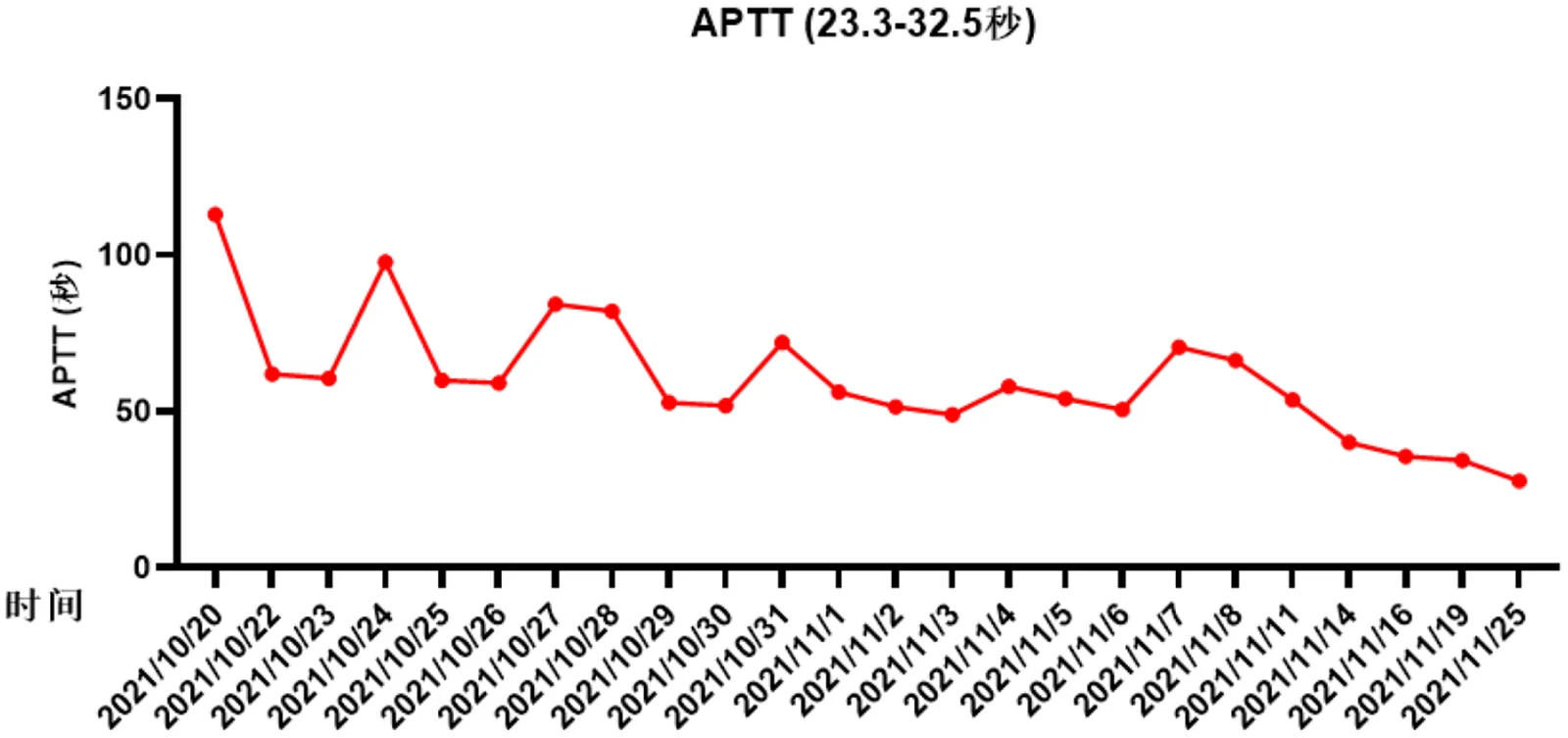
Trend of APTT changes during hospitalization (normal range 23.3–32.5 seconds). APTT = activated partial thromboplastin time.

Trend data of APTT changes during hospitalization indicated that, with administration of human prothrombin complex for hemostasis, dexamethasone combined with cyclophosphamide for autoantibody suppression, and doxycycline for treatment of the Flavobacterium odoratum-like bacterial infection, the patient’s APTT gradually decreased from 113.1 seconds at admission to 27.7 seconds, which falls within the normal reference range of 23.3 to 32.5 seconds (Fig. 1).

Data on FVIII activity and FVIII antibody levels during hospitalization showed that FVIII activity increased from 0.7% at admission to 116.1%, remaining stably within the normal range of 70–120%. Concurrently, FVIII antibody levels declined from 1.7 BU/mL at admission to 0 BU/mL and remained within the normal range of 0–0.6 BU/mL (Table 1).

**Table 1:** Trend of changes in FVIII activity and FVIII antibody.

Date	FVIII activity (70%–120%)	FVIII Antibody (0–0.6)
October 20, 2021	0.7	1.7
November 04, 2021	1.8	–
November 25, 2021	52.2	–
November 02, 2021	116.1	0

Results of urine quantitative culture and drug susceptibility testing during hospitalization confirmed infection with *F.odoratum*, which exhibited resistance to multiple antimicrobial agents including piperacillin, piperacillin/tazobactam, cefazolin, ceftazidime, ceftriaxone, cefepime, aztreonam, imipenem, meropenem, gentamicin, amikacin, tobramycin, levofloxacin, and ciprofloxacin (Table 2).

**Table 2:** Urine quantitative culture + drug sensitivity test results.

Antibacterial drugs	MIC (Minimum inhibitory concentration mg/L)	Sensitivity
Piperacillin	>=128	Drug-resistant
Piperacillin/Tazobactam	>=128	Drug-resistant
Cefazolin	>=64	Drug-resistant
Ceftazidime	>=64	Drug-resistant
Ceftriaxone	>=64	Drug-resistant
Cefepime	>=64	Drug-resistant
Aztreonam	>=64	Drug-resistant
Imipenem	>=16	Drug-resistant
Meropenem	>=16	Drug-resistant
Gentamicin	>=16	Drug-resistant
Amikacin	>=64	Drug-resistant
Tobramycin	>=16	Drug-resistant
Levofloxacin	>=8	Drug-resistant
Ciprofloxacin	>=4	Drug-resistant
Cotrimoxazole	>=16/304	Drug-resistant

## 4. Discussion

AHA is a rare but severe autoimmune bleeding disorder, with an estimated annual incidence of 1 to 1.5 cases per million population.^[9]^ It may occur at any age and affects both sexes equally. Two peaks in incidence have been observed: among individuals over 60 years of age and among women during the peripartum period. The underlying etiology of AHA remains largely unknown and may be associated with malignancies, hematologic neoplasms, autoimmune diseases, dermatologic conditions, drug exposure, pregnancy, and, more recently reported, infections such as SARS-CoV-2, COVID-19 vaccination, and Helicobacter pylori infection.^[3,10,11]^ Therefore, comprehensive evaluation to identify potential triggering factors is essential upon diagnosis. To our knowledge, this is the first documented case of *F.odoratum* infection associated with AHA.

@@43@@F. *odoratum* is an aerobic, Gram-negative bacillus and a conditional pathogen, with an extremely low isolation rate in clinical samples.^[12]^ This bacterium exhibits strong adhesive properties and can be moderately invasive, particularly in individuals with impaired immune function, including the elderly, those with immunocompromised conditions, long-term users of broad-spectrum antibiotics, and patients with indwelling urinary catheters. Once detected, it may lead to life-threatening infections.^[13–19]^ Following infection, the primary clinical manifestations include urinary tract infections, bloodstream infections, skin and soft tissue infections, respiratory tract infections, and central nervous system infections. Given this clinical context, the infection is likely associated with the persistent presence of the urinary catheter. Spanik et al^[18]^ further suggested that long-term urinary catheterization may represent an independent risk factor for such bacterial infections. Therefore, the prolonged catheterization in this patient indicates that this intervention constitutes a significant risk factor for infection. Additionally, the patient’s history of ureterolithotomy and double-J stent placement suggests that these procedures may create potential pathways for bacterial colonization and subsequent infection.

Currently, the pathogenesis of AHA remains unclear. In recent years, some studies have proposed surgery as a potential trigger for AHA,^[20]^ with the underlying mechanism involving tissue injury-induced activation of coagulation factors along the coagulation cascade, leading to the production of autoantibodies against specific coagulation factors and thereby precipitating AHA. In this case, the patient developed AHA following prior ureteral stone removal and double-J stent insertion: procedures associated with surgical tissue trauma. Research by Liu Ling and colleagues further supports that surgical injury may act as a triggering factor for AHA.^[21,22]^ Recently, Japanese scholars reported a case of AHA complicated by Helicobacter pylori infection leading to an infectious aortic aneurysm. The proposed pathogenesis of AHA in such cases involves infection-mediated disruption of immune tolerance to endogenous factor VIII. One explanatory framework is the danger signal theory, which posits that tissue damage or inflammatory cell injury disrupts self-immune tolerance, resulting in the production of anticoagulant autoantibodies and the onset of AHA. Similarly, in the present case, *F.odoratum* infection may have disrupted autoimmune tolerance through tissue invasion, promoting the generation of abnormal coagulation factor inhibitors and ultimately triggering AHA. These findings collectively suggest that both invasive surgical procedures and *F.odoratum* infection may serve as etiological contributors to AHA.

Due to the multidrug-resistant characteristics of *F.odoratum* bacteria, clinical treatment has been significantly challenged. In this case, 2 urine quantitative cultures and antimicrobial susceptibility tests from the patient both confirmed infection with *F.odoratum* organisms, which exhibited resistance to piperacillin, piperacillin/tazobactam, cefazolin, ceftazidime, ceftriaxone, cefepime, aztreonam, imipenem, meropenem, gentamicin, amikacin, tobramycin, levofloxacin, and ciprofloxacin. According to published literature, doxycycline,^[8]^ minocycline,^[23]^ and rifampicin^[16]^ have demonstrated efficacy against infections caused by Scallotropha species. Based on available evidence, doxycycline was selected for treatment. Following continuous bladder irrigation and rational antibiotic administration, repeat urine culture after 10 days showed normalization.

Currently, management of AHA includes addressing underlying conditions, controlling acute bleeding episodes, and eliminating autoantibodies.^[24]^ For hemostatic therapy, recombinant activated factor VII and activated prothrombin complex concentrate are considered first-line treatments for bleeding in AHA patients.^[1]^ The choice of hemostatic agent depends on both the severity of bleeding and the titer of factor VIII inhibitors. Patients with factor VIII inhibitor levels <5 BU may respond to human factor VIII concentrate, whereas those with levels >5 BU typically require bypassing agents such as activated prothrombin complex concentrate or activated factor VII. A retrospective study of 34 AHA patients demonstrated the effectiveness of human prothrombin complex concentrate, reporting an overall response rate of 86%, with a standard dosing regimen of 75 U/kg every 8 to 12 hours.^[25]^ In this case, the patient was treated with human prothrombin complex concentrate, leading to successful control of hematuria and subcutaneous hematoma. Autoantibody clearance relies on immunosuppressive therapy.^[25]^ Various regimens have been reported, including corticosteroids alone or in combination with cytotoxic agents (e.g., cyclophosphamide, azathioprine, 6-mercaptopurine, vincristine), rituximab, or other immunomodulatory drugs. Evidence suggests that combination therapy with corticosteroids and cyclophosphamide is superior to corticosteroid monotherapy.^[26]^ This patient received dexamethasone combined with cyclophosphamide, resulting in dynamic improvements: prolonged APTT shortened, factor VIII activity gradually increased, clinical symptoms resolved, coagulation parameters normalized, factor VIII inhibitors were eradicated, and ultimately, disease remission was achieved.

## 5. Conclusion

AHA is a rare but potentially life-threatening hemorrhagic disorder, where delayed diagnosis and inadequate management are major contributors to high mortality. Early recognition and prompt initiation of appropriate therapy are critical to reducing mortality and improving outcomes. To date, there have been no clinical reports of concurrent infection with *F. odoratum* and AHA. There is a lack of mature diagnostic and therapeutic experience for such diseases. In this case, the patient was treated with a comprehensive treatment plan, achieving relatively satisfactory therapeutic effects. This study provides an initial exploration and experience accumulation for the diagnosis and treatment of this disease. At the same time, clinicians should be aware that bacterial infections, particularly in patients with long-term indwelling catheters, may trigger the development of AHA. Timely selection of effective antibiotics and early diagnosis of AHA are essential components of optimal patient management.

## Acknowledgments

The authors gratefully acknowledge the patient for making this case report possible, as well as all healthcare professionals and researchers involved in this study.

The study was approved by Zunyi Medical University Hospital’s Ethics Committee. It was a retrospective case report using routine clinical data with no additional interventions. The patient provided written informed consent for publication.

## Author contributions

**Project administration:** Mingqiang Ren.

**Writing – original draft:** Di Chen.

**Writing – review & editing:** Ping Yuan.
